# COVID-19 variants as moving targets and how to stop them by glycoengineered whole-virus vaccines

**DOI:** 10.1080/21505594.2021.1939924

**Published:** 2021-07-25

**Authors:** Uri Galili

**Affiliations:** Department of Medicine, Rush Medical College, Chicago, IL, USA

The use of gene-based Covid-19 vaccines containing the S protein gene has proven to be very effective in protection against infection by SARS-CoV-2. However, increasing numbers of variants with higher transmissibility and/or virulence have been reported [1,2]. These variants raise concerns that immunization with gene-based vaccines containing only the S protein gene may allow for emergence of more virulent mutated variants by a Darwinian-like “natural-selection” process. Such variants may evade protective anti-S protein antibodies in immunized individuals. A concrete example for a virus acquiring mutations that facilitate immune evasion in humans (called “escape-mutations”) is that of HIV within infected patients [3]. These mutations increase the number of N (asparagine)-linked carbohydrate chains (N-glycans) forming the “glycan-shield” on gp120 of HIV. Gp120 is the envelope glycoprotein that mediates HIV infection of host-cells, analogous to the role of S protein in SARS-CoV-2. The glycan-shield on gp120 masks many antigenic epitopes on this glycoprotein [4] and the additional glycans on mutated HIV variants prevent binding of neutralizing-antibodies [3]. The S protein of SARS-CoV-2 has a glycan-shield of 22 N-glycans, covering ~65% of its surface and thus masking various antigenic peptides from detection by host immune systems [4,5]. N-glycans are synthesized on asparagine (Asn) within the Asn-X-Ser/Thr (N-X-S/T) sequon, where X is any amino acid other than proline. Thus, any substitution mutation that introduces Asn or Ser/Thr to form this sequon will result in synthesis of additional N-glycans that can further mask regions of the S protein recognized by neutralizing antibodies.

Reviewing of the amino acid (aa) sequence of the S1 subunit of the S protein (14–685 residues responsible for receptor binding) indicates that there are 40 Asn not within sequons. Substitution-mutations resulting in appearance of Ser or Thr as the second downstream aa will form the sequon yielding a new N-glycan. In addition, there are 46 Ser and 41 Thr. Substitution-mutations resulting in appearance of Asn as the second upstream aa will also form escape-mutation sequons carrying N-glycans that mask antigenic peptides. These suggest that the probability for appearance of variants with new N-glycans is not negligible.

Additional kinds of escape-mutations in the S protein gene which change protein structure, also may alter the antigenicity of the S protein thus affecting the ability of the immune system to neutralize such variants in individuals immunized by this protein. A recent study demonstrating such escape-mutations reported on the appearance of such variants in patients with long-term SARS-CoV-2 infections. These mutations are characterized by in-frame 3, 6, 9, and 12 aa deletions in the N-terminal domain (NTD) and are resistant to monoclonal anti-S protein neutralizing antibodies, but not to polyclonal antibodies in the serum of convalescent individuals [6].

A plausible scenario leading to appearance of a SARS-CoV-2 variant capable of evading the post-vaccination protective immune response is as follows: In the course of virus replication in a non-immunized individual, multiple random mutations occur. A rare mutation(s) in the S protein gene may be an escape-mutation resulting in synthesis of an additional glycan(s) that masks the S protein from neutralizing antibodies [3] in a very small proportion of the virions. Alternatively, the mutation may be a deletion in the NTD which confers resistance to anti-S protein neutralizing antibodies [6]. Upon transmission of viruses from such a non-immunized infected individual to an immunized unprotected individual (i.e., not using appropriate mask and not keeping social distance), few virions with the escape-mutation will be transmitted together with the many non-mutated virions. In the infected immunized individual, the non-mutated virions will be neutralized by the protective anti-S protein antibodies. The absence of the non-mutated virus will lead to selective replication and expansion of the mutated virus which evades the neutralizing anti-S protein antibodies. This selective expansion will result in the appearance of a new resistant variant that can spread throughout populations which were vaccinated with the S protein gene-based vaccine. This scenario may repeat itself because new mutations keep emerging in replicating SARS-CoV-2, even when a new vaccine, adapted to immunize against the mutated virus, is used. Such mutations make Covid-19 variants moving targets difficult to eradicate by any single-antigen vaccine. Similar considerations apply to future gene-based single-antigen vaccines against other viruses, as well.

Based on these considerations it is suggested that second generation Covid-19 vaccines should include several viral antigens (multi-antigenic vaccines), so that virions carrying mutations that enable evasion from the anti-S protein immune response will be destroyed by the immune response to other vaccinating viral antigens. A simple approach for preparation of multi-antigenic vaccines is to use all viral proteins as vaccinating antigens in the form of inactivated SARS-CoV-2 whole-virus vaccine, provided that these antigens are of high immunogenicity. SARS-CoV-2 whole-virus vaccine is currently produced by propagation of the virus in Vero cells [7]. Recent reports in public media from some of the countries using this vaccine have indicated, however, that the efficacy of this vaccine is variable. One reason for suboptimal vaccine efficacy is the SARS-CoV-2 glycan-shield which masks various antigenic peptides from detection by host immune systems [4,5] and which causes electrostatic repulsion from antigen-presenting-cell (APC) surface by negatively charged sialic acid units both on glycan-shields and APC [8]. The present manuscript proposes simple methods for increasing the immunogenicity of SARS-CoV-2 whole-virus vaccine by engineering glycans (glycoengineering) to convert the glycan-shield that masks the virus, into a glycan-shield that actively targets the vaccinating virus for extensive uptake by APC such as dendritic-cells and macrophages. This targeting greatly increases the amount of vaccinating virus that is processed and presented by APC for T cell activation, thereby amplifying vaccine efficacy.

Targeting vaccinating virus to APC is achieved by glycoengineering the glycan-shield of the S protein on the virus to present multiple α-gal epitopes (Galα1-3Galβ1-4GlcNAc-R). These vaccines (referred to as SARS-CoV-2_α-gal_) form immune-complexes at the vaccination site with the abundant natural anti-Gal antibody (~1% of circulating immunoglobulins in humans) [9] which binds specifically to α-gal epitopes [10,11]. It is suggested that anti-Gal/SARS-CoV-2_α-gal_ immune-complexes will be targeted for extensive endocytosis by APC following binding of the Fc “tail” of the immunocomplexed anti-Gal to Fcγ receptors on APC (Figure 1). The APC will further transport the large amounts of internalized vaccinating virus to regional lymph-nodes, process and present virus antigenic peptides that will effectively activate many more T-cells than vaccination with SARS-CoV-2 lacking α-gal epitopes. Helper T-cells activated by SARS-CoV-2_α-gal_ will activate many more B-cells producing anti-SARS-CoV-2 antibodies than following activation with non-immunocomplexed SARS-CoV-2 vaccine. Similarly, many more cytolytic T-cells (CTL) will be activated by SARS-CoV-2_α-gal_ vaccine than by SARS-CoV-2 and thus increase the ability to destroy cells infected by the virus. The increased numbers of virus specific activated T and B-cells will prolong the period of active immune protection and improve the efficacy of the protective immune memory than the corresponding cells activated by vaccinating SARS-CoV-2 virus that is randomly internalized by APC. Thus, it is suggested that SARS-CoV-2_α-gal_ vaccine will induce better and longer protection than the currently used SARS-CoV-2 vaccines which lack α-gal epitopes.

**Figure 1.**
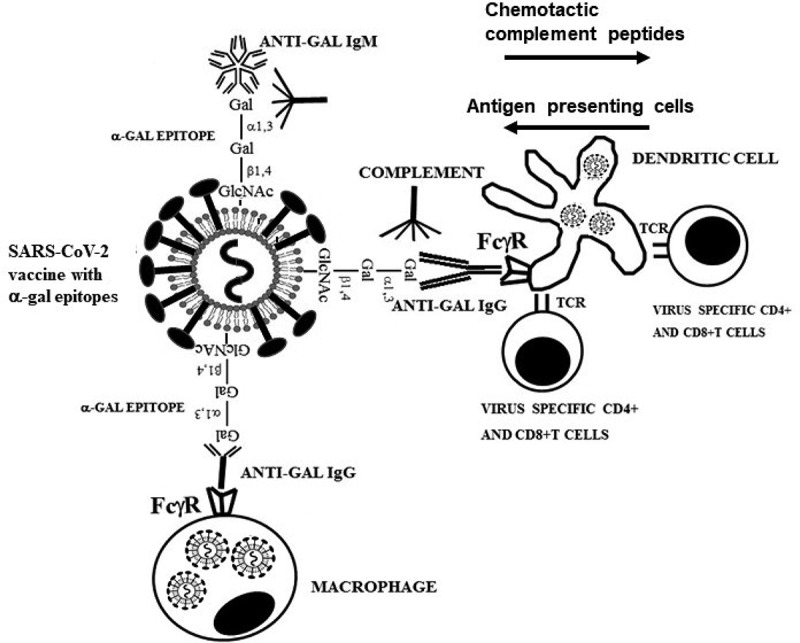
Hypothesis on harnessing of the natural anti-Gal antibody for increased efficacy of inactivated SARS-CoV-2_α-gal_ whole-virus vaccine. Vaccinating SARS-CoV-2_α-gal_ binds the natural anti-Gal antibody at the vaccination site and activates the complement system to generate complement cleavage chemotactic peptides that recruit APC such as dendritic cells and macrophages. Anti-Gal/SARS-CoV-2_α-gal_ immune-complexes are targeted for active, extensive uptake by APC, following binding of the Fc portion of immunocomplexed anti-Gal to Fcγ receptors (FcγR) on APC. Such uptake may be mediated also by C3b/CR1 interaction. The APC transport internalized SARS-CoV-2_α-gal_ to regional lymph nodes, process and present the immunogenic viral peptides on class-I and class-II MHC molecules for activation of virus-specific CD8^+^ and CD4^+^ T cells, respectively. TCR-T-cell receptors. Modified with permission from Galili U. “*The natural anti-Gal antibody as foe turned friend in medicine*” Academic Press/Elsevier, London, 2018, page 153

The extent of anti-Gal mediated increased viral vaccine efficacy has been demonstrated in mice producing anti-Gal and vaccinated with inactivated influenza-virus glycoengineered to present multiple α-gal epitopes on the hemagglutinin (HA) envelope glycoprotein (influenza-virus_α-gal_). Influenza-virus_α-gal_ vaccine induced anti-virus antibody production at titers ~100-fold higher than influenza-virus vaccine lacking α-gal epitopes. T-cell response was 6–100-fold higher and survival following challenge with a lethal dose of influenza-virus was ~90% vs. ~10%, respectively [12]. A similar increase in antibody and T-cell response, as well as in HIV neutralizing activity of antibodies, was observed in anti-Gal producing mice immunized with recombinant gp120 of HIV vaccine glycoengineered to present α-gal epitopes (i.e., gp120_α-gal_), in comparison to a similar vaccine lacking these epitopes [13]. Overall, these studies suggest that immunization with SARS-CoV-2_α-gal_ whole-virus vaccine may have a similar marked increased efficacy in comparison to available SARS-CoV-2 whole-virus vaccine lacking α-gal epitopes.

Since some whole-virus vaccines display suboptimal immunogenicity, there is a general concern that such vaccines may induce production of antibodies mediating antibody-dependent enhancement (ADE) of virus infection [14,15]. Antibodies mediating ADE have low affinity, thus they fail to neutralize the virus, but mediate uptake of the virus via Fc/Fc receptor interaction. ADE mediates uptake of the virus by cells with Fc receptors, such as macrophages, without destruction of the virus. The internalized virus replicates within the cells, thereby increasing the infection of hosts [14,15]. It is unlikely that ADE will occur following vaccination with SARS-CoV-2_α-gal_. This assumption is based on the observations of 100-fold higher antibody response following immunization with influenza-virus_α-gal_ [12] and with gp120_α-gal_ [13], in comparison to virus vaccines lacking α-gal epitopes, near complete protection against infection with a lethal dose of influenza-virus in mice immunized with influenza-virus_α-gal_ [12] and effective production of neutralizing anti-HIV antibodies in mice immunized with gp120_α-gal_, but not with gp120 [13]. Nevertheless, analysis of ADE should be performed in pre-clinical studies with SARS-CoV-2_α-gal_ to confirm the absence of this phenomenon following vaccination with the glycoengineered virus.

Synthesis of α-gal epitopes on SARS-CoV-2 is catalyzed by the glycosylation enzyme α1,3galactosyltranferase (α1,3GT) which is naturally active in nonprimate-mammals, lemurs and New-World monkeys [16]. Three of the methods for synthesis of α-gal epitopes on the glycan-shield of SARS-CoV-2 were previously described in detail [8] and are: 1. Enzymatic replacement of sialic acid on the glycan-shield with α-gal epitopes, by combined activities of neuraminidase (for removal of sialic acid), recombinant α1,3GT and uridine-diphosphate-galactose (UDP-Gal) as sugar donor. Such enzymatic reaction was used for glycoengineering of gp120 into gp120_α-gal_ [13], whereas conversion of influenza-virus into influenza-virus_α-gal_ required the use of only recombinant α1,3GT and UDP-Gal, since the virus lacks sialic acid [12]. 2. Propagating SARS-CoV-2 in Vero cells (or other host-cells) engineered to produce large amounts of α1,3GT by stable-transfection with several copies the *α1,3GT* gene (*GGTA1*). 3. Transduction of host-cells with replication-defective adenovirus containing the *α1,3GT* gene, prior to host-cell infection with replicating SARS-CoV-2 for vaccine preparation. Such transduction introduces multiple (~20) copies of the *α1,3GT* gene per cell [17]. Subsequent infection of host-cells with SARS-CoV-2, will result in synthesis of multiple α-gal epitopes on the replicating virus by α1,3GT encoded by the *α1,3GT* gene introduced into the host-cells by the replication defective adenovirus.

In conclusion, the use of multi-antigenic Covid-19 vaccines will prevent the risk for “evolution” of variants that are resistant to the protective immune response elicited by gene-based vaccines containing only the S protein gene. It is suggested that inactivated SARS-CoV-2 whole virus could serve as effective multi-antigenic vaccine if it is glycoengineered to present multiple α-gal epitopes in order to achieve anti-Gal mediated targeting to APC for extensive uptake of the vaccinating virus by these cells. Since few individuals produce anti-Gal IgE causing meat allergy to α-gal epitopes in beef, pork and lamb (“α-gal syndrome”) [18,19], those with this syndrome should receive vaccines presenting α-gal epitopes in clinics equipped for treating allergic reactions.

## Data Availability

Data mentioned in this commentary are included in the following manuscripts which are available as free PMC articles in PubMed (https://pubmed.ncbi.nlm.nih.gov/)-
Abdel-Motal U, Wang S, Lu S, Wigglesworth K, Galili U. Increased immunogenicity of human immunodeficiency virus gp120 engineered to express Galalpha1-3Galbeta1-4GlcNAc-R epitopes. J Virol. 2006 Jul;80(14):6943-51. doi: 10.1128/JVI.00310-06. PMID: 16809300; PMCID: PMC1489031. (reference 13 in this commentary). Abdel-Motal UM, Guay HM, Wigglesworth K, Welsh RM, Galili U. Immunogenicity of influenza virus vaccine is increased by anti-gal-mediated targeting to antigen-presenting cells. J Virol. 2007 Sep;81(17):9131-41. doi: 10.1128/JVI.00647-07. Epub 3 July 2007. PMID: 17609270; PMCID: PMC1951452. (reference 12 in this commentary).
